# Systematic network lesioning reveals the core white matter scaffold of the human brain

**DOI:** 10.3389/fnhum.2014.00051

**Published:** 2014-02-11

**Authors:** Andrei Irimia, John D. Van Horn

**Affiliations:** Department of Neurology, Keck School of Medicine, Institute for Neuroimaging and Informatics, University of Southern CaliforniaLos Angeles, CA, USA

**Keywords:** connectomics, traumatic brain injury, brain network, neurotrauma, neuroimaging, MRI, DTI

## Abstract

Brain connectivity loss due to traumatic brain injury, stroke or multiple sclerosis can have serious consequences on life quality and a measurable impact upon neural and cognitive function. Though brain network properties are known to be affected disproportionately by injuries to certain gray matter regions, the manner in which white matter (WM) insults affect such properties remains poorly understood. Here, network-theoretic analysis allows us to identify the existence of a macroscopic neural connectivity core in the adult human brain which is particularly sensitive to network lesioning. The systematic lesion analysis of brain connectivity matrices from diffusion neuroimaging over a large sample (*N* = 110) reveals that the global vulnerability of brain networks can be predicated upon the extent to which injuries disrupt this connectivity core, which is found to be quite distinct from the set of connections between rich club nodes in the brain. Thus, in addition to connectivity within the rich club, the brain as a network also contains a distinct core scaffold of network edges consisting of WM connections whose damage dramatically lowers the integrative properties of brain networks. This pattern of core WM fasciculi whose injury results in major alterations to overall network integrity presents new avenues for clinical outcome prediction following brain injury by relating lesion locations to connectivity core disruption and implications for recovery. The findings of this study contribute substantially to current understanding of the human WM connectome, its sensitivity to injury, and clarify a long-standing debate regarding the relative prominence of gray vs. WM regions in the context of brain structure and connectomic architecture.

## Introduction

Brain lesions due to conditions such as traumatic brain injury (TBI), stroke and multiple sclerosis (MS) can have focal, region-specific consequences as well as diffuse effects upon cortical circuitry (Van Horn et al., 2012). For this reason, the ability to quantify injury-related connectomic changes in a systematic manner is critical for the evaluation of injury severity and for the personalization of treatment after neurotrauma. In both health and disease, network theory can provide essential insight into the structural properties of brain connectivity (Sporns, 2011), particularly by providing quantitative measures of lesion impact upon neural structure and function, with possible relevance to the prediction of clinical outcome variables and to the task of designing patient-tailored rehabilitation protocols (Irimia et al., 2012a,b).

Within the modeling framework of network theory, the gray matter (GM) of the brain can be parcellated into distinct regions which are conceptualized as graph nodes connected by edges whose properties are specified by white matter (WM) connections, having complex topological relationships within the hierarchy of the network. Investigating network integration and segregation using network theory allows one to quantify how much information brain regions can exchange as well as the extent to which such regions remain structurally segregated from each other (Sporns, 2011). On the one hand, network integration captures the capacity of a network to engage in global interactions which transcend the boundaries of local network modules so as to enable network-wide integration; on the other hand, segregation quantifies effective changes in the strength of interactions as nodes become more topologically remote from each other (Rubinov and Sporns, 2010). Because network integration and segregation reflect network vulnerability to insult, these essential network properties can aid one to understand the effect of injury upon the brain.

Recent advances in connectomic and network theoretic analysis have led to an improved understanding of how GM regions are organized from the standpoint of their ability to communicate. For example, it has been proposed that the human connectome has a “rich club” organization, where high-degree cortical nodes are more densely connected to each other than to nodes of lower degree (Van Den Heuvel and Sporns, 2011). In comatose TBI patients, network node topological properties have been found to reorganize themselves radically as a consequence of injury, with theoretical implications for models of consciousness and practical ones for clinical care (Achard et al., 2012). Damage to brain regions important for communication between functional sub-networks has been found to decrease network modularity as computed based on functional magnetic resonance imaging (fMRI) recordings (Gratton et al., 2012). Similarly, one study on stroke patients found that peri-lesional circuits have reduced abilities to communicate with the rest of the brain (Crofts et al., 2011). Such findings reflect appreciable efforts dedicated to understanding the properties of brain network cortical *nodes*, though less emphasis has been placed on determining the properties and differential prominence of network WM *edges* based on their contributions to network integration.

By studying how brain vulnerability to insult varies as a function of WM and GM injury location, lesion effects upon network properties can be assessed. In this study, we investigate the effects of both localized and diffuse injury upon the network properties of the human connectome using models of brain connectivity based on MRI and diffusion tensor imaging (DTI). By further combining MRI and DTI analysis methods with connectomics and network theory, we identify the existence of a macroscopic neural connectivity core in the human brain. This subset of WM pathways has properties which are particularly important to inter-regional connectivity and it is found that injury to the connectomic core substantially affects brain network organization. Importantly, we propose that the WM connectivity scaffold of network edges stands in complement to the rich club of nodes in brain networks, leading to a relationship of structural complementarity between important WM fibers and prominent GM regions, respectively. We justify this conclusion based on a direct comparison between the rich club network of the brain and its connectivity scaffold, which are found to differ appreciably. The nature of the complementary relationship between the rich club network and the connectivity scaffold contributes essential information to the long-standing debate regarding the relative prominence of GM vs. WM regions within human brain architecture. An important strength of the present study is that it quantifies the connectomic core using a population sample of larger size (*N* = 110) than typically used in previous connectomic studies. Aside from contributing substantially to present understanding of the human connectome, this study bears special significance upon network theory use to understand the effects of neurotrauma. Specifically, the systematic lesion analysis demonstrated here reveals that brain network vulnerability is largely dependent upon connectivity core disruption, which can provide appreciable insight on how to integrate computational models of traumatic lesions with existing protocols for brain lesion assessment, rehabilitation and clinical care.

## Materials and methods

### Subjects and data acquisition

We used *T*_1_-weighted structural MRI volumes acquired from *N* = 110 healthy, right-handed human males aged 25–36 (mean: 30 years; standard deviation: 3.18 years) whose neuroimaging data were stored in the Integrated Data Archive (IDA, ida.loni.usc.edu) of the Laboratory of Neuro Imaging (LONI) and Institute for Neuroimaging and Informatics (INI) at the University of Southern California. Subjects provided their informed written consent as required by the Declaration of Helsinki, U.S. 45 CFR 46, and neuroimage volume acquisition was conducted with the approval of local ethics committees at the respective research institutions where data were acquired. Subjects were all healthy and had no history of neurological or psychiatric illnesses. Neuroimaging data sets in the LONI IDA are fully anonymized for such purposes as sharing, re-use, and re-purposing, and no linked coding or keys to subject identity are maintained. For these reasons, in compliance with the U.S. Health Insurance Portability and Accountability Act (HIPAA; http://www.hhs.gov/ocr/privacy), this study does not involve human subjects' materials. Both structural MRI and DTI volumes were acquired at 3 T using a Siemens Magnetom TrioTim MRI scanner. For the MRI volumes, an MP-RAGE sequence was used (voxel size: 1 × 1 × 1 mm; *TR* = 1900 ms; *TE* = 2.26 ms; *TI* = 900 ms; flip angle: 9°). For DTI, volumes were acquired in 64 gradient directions (voxel size: 2 × 2 × 2 mm; *TR* = 7000 ms; *TE* = 93 ms).

### Image processing

The LONI Pipeline environment (http://pipeline.loni.usc.edu) was employed for all major image processing operations, including bias field correction, skull stripping, image alignment, etc. This program is a graphical environment for the construction, execution and validation of neuroimaging data analysis and facilitates automated data format conversion while providing a large library of computational tools (Mackenzie-Graham et al., 2008; Dinov et al., 2009, 2010). DTI data were analyzed in native subject space using TrackVis (http://trackvis.org) to reconstruct fiber tracts via deterministic tractography, which was used instead of probabilistic tractography because the latter is often perceived to be a more standard, more commonly used and better-understood methodology. Eddy current correction was applied to the DTI volumes using the FSL FLIRT utility (http://fsl.fmrib.ox.ac.uk/fsl), and the Diffusion Toolkit within TrackVis was used for DTI reconstruction, fiber tracking and spline filtering. For each subject, the skull was stripped using the skull-stripping utility of the AFNI package (Smith, 2002) and bias field corrections were applied to the MRI volumes using tri-cubic B spline interpolation. FreeSurfer (FS) software was used to perform segmentation and regional parcellation (Fischl et al., 1999a, 2002) according to methodologies described by Destrieux et al. (2010). MRI and DTI volumes were co-registered before further analysis. Processing workflows to compute inter-regional connectivity matrices were constructed using purpose-built software whose reliability analysis is described elsewhere (Irimia et al., 2012c). For each hemisphere, 74 cortical structures were identified in addition to 7 subcortical structures and to the cerebellum. One midline structure (the brain stem) was also included, for a total of 165 parcels for the entire brain. The cortex was divided into 7 lobes, with the number of parcels in each being equal to 21 (frontal, Fro), 8 (insula, Ins), 8 (limbic, Lim), 11 (temporal, Tem), 11 (parietal, Par), 15 (occipital, Occ).

### Connectivity calculation

To compute connectivity between regions for each subject, the location of each fiber tract extremity within the brain was identified, while the GM volume associated with each parcel was also delineated. For those fibers which both originated as well as terminated within any two distinct parcels of the 165 available, each fiber extremity was associated with the appropriate parcel. For each such fiber, the corresponding entry in the connectivity matrix of the subject's brain was appropriately updated to reflect an increment in fiber count (Hagmann et al., 2008, 2010) to generate a connectivity matrix with entries specifying the total fiber count between each pair of regions. Fibers with lengths shorter than 1.5 cm were discarded. Each subject's connectivity matrix was normalized over the total number of fibers within that subject; for population-level analysis, all connectivity matrices were pooled across subjects and averaged to compute probabilistic connectivity values. The average length of the fibers connecting every pair of regions was also recorded, as was the average fractional anisotropy (FA) of each fiber line as reconstructed via tractography. Processing workflows to compute inter-regional connectivity matrices were constructed using purpose-built software.

### Connectogram design

**Figure 1:**
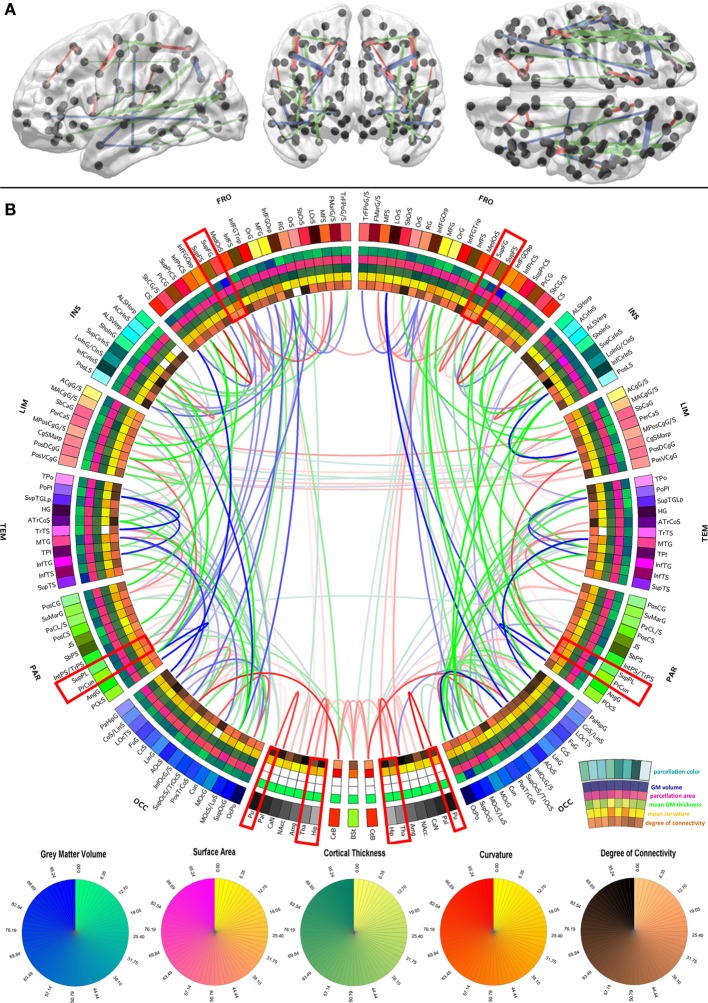
**Graphical representation of the human brain connectivity scaffold**. Standard 3D graphs **(A)** and a connectogram **(B)** are used to visualize WM connections whose removal leads to significant changes in network integration and segregation. Only connections with this property are represented, and the strength of the link associated with each of them is indicated by the *F* statistic of the test. Link transparency varies such that most transparent links are those associated with the smallest *F*-values, and the most opaque ones are associated with the largest *F*-values (see section Statistical significance of edge removals for details). To facilitate visibility of the most prominent core connections, the significance threshold used for 3D graphs (α/*m* = 7.4 × 10^−9^ where α = 0.0001 is the statistical significance level, *m* = *G* × (G − 1)/2 is the number of comparisons, and *G* = 165 is the number of parcels) is more stringent than for the connectogram (α/*m* = 3.7 × 10^−6^ where α = 0.05). The significance threshold used for 3D graphs **(A)** is more stringent than for the connectogram **(B)** in order to facilitate visibility of the most prominent core connections **(A)**, as opposed to all the connections whose removal leads to statistically significant changes in network integration and segregation **(B)**. Regions whose connectogram wedges are highlighted in red correspond to rich club nodes as identified by Van Den Heuvel and Sporns (2011); the number of core scaffold connections and the complexity of their pattern compared to rich club interlinks both suggest considerable differences between the rich club network and the core scaffold (see discussion). For a connectogram where network metrics—rather than morphometric variables—are encoded in each concentric circle, see our previous publication (Van Horn et al., 2012).

Connectivity was represented circularly using a framework based on Circos software (Krzywinski et al., 2009). Parcellated regions were displayed as a circle of radially aligned elements (a connectogram) representing the left and right hemispheres positioned symmetrically on the corresponding side of the vertical axis. Parcellated regions were assigned unique RGB colors listed exhaustively elsewhere (Irimia et al., 2012c). Arrangement of parcellations within each lobe of the connectogram was performed in the order of their locations along the antero-posterior axis of the cortical surface associated with the published FS normal population atlas (Destrieux et al., 2010). Cortical lobes were assigned unique color schemes: black to red to yellow (Fro), charlotte to turquoise to forest green (Ins), primrose to lavender rose (Lim), etc. Subcortical structures were colored light gray to black. An unambiguous abbreviation scheme was created to label each parcellation, as summarized in Irimia et al. (2012c). Within the outermost circle which represents cortical parcellations, five circular heat maps were created to encode one of the five structural measures associated with the corresponding parcellation. Proceeding toward the center of the circle, these measures are total GM volume, total area of the surface associated with parcellation, mean cortical thickness, mean curvature and connectivity per unit volume. This latter measure was calculated as the density of fibers with endings within that parcellation divided by the parcellation's total GM volume. The value of each structural measure was encoded as a color using a color scheme mapping that ranged from the minimum to the maximum of the data set. Specifically, the cortical thickness *t* with values in the interval (*t*_min_, *t*_max_) was normalized as *t*_1_ = (*t* − *t*_min_)/(*t*_max_ − *t*_min_). The value of *t*_1_ was associated with a unique color; for example, nuances at the extremities of the color map correspond to *t*_min_ and *t*_max_, as required. For the brain stem, cerebellum and subcortical structures, values for area, thickness and curvature were unavailable from FS and their appropriate heat map entries were drawn in white. The methodology for generating connectograms and guidelines for their interpretation are also described elsewhere (Irimia et al., 2012c); see also the caption to Figure 1.

### Network measures

Network metrics were computed for each subject, starting with the *degree* of each node. Here, nodes are denoted by parcellated regions and edges are represented by fiber tracts. The node degree is the number of edges connected to a node and its calculation has fundamental impact upon many network measures; moreover, node degree distributions are highly informative of network architecture. The entry indexed by *i* and *j* in the *distance* matrix of the graph contains the minimum length of the path connecting vertices *v*_*i*_ and *v*_*j*_ and was computed using the algebraic shortest paths algorithm implemented by Rubinov and Sporns (2010) for the purpose of studying brain networks. These authors proposed that a number of prominent network properties can be divided into two important families (measures of integration vs. segregation), and the values of measures in each of these two families are in fact often correlated (Van Horn et al., 2012). For this reason, we have opted to focus here on one representative measure of each type when studying the effect of GM region removal. To study network integration, the *characteristic path length* λ of the network was computed, which is the global average of the entries in the distance matrix. The lower λ is, the lower the average cost of reaching a node from any other node. To investigate local network segregation, the average *local efficiency* of the network was calculated. For a node, the local efficiency is the average of the inverse entries of the connectivity matrix computed on the neighborhood of the node. For a network, the average local efficiency of the network is the mean value of nodal local efficiency computed over all nodes.

It is important to clarify that this study attempts to investigate two distinct types of network lesions, namely (A) the removal of network edges (which represent WM connections between pairs of nodes), and (B) the removal of network nodes (which represent gyri and sulci). Additionally, the study seeks to quantify the effect of such removals *upon the entire network* in order to determine which lesions have global-level effects upon the brain. For these two reasons, the metrics which were selected for investigating the effects of GM region removals are λ and the average local efficiency, because these metrics describe global changes to the network, as elaborated by Rubinov and Sporns (2010). Network measures of interest which were included in the analysis of edge removals included *assortativity* (Newman, 2002) (the correlation coefficient for the degrees of neighboring nodes in the network), *graph diameter* (the largest entry in the graph distance matrix), *eccentricity* (the greatest geodesic distance between any two vertices in the graph), *radius* (the minimum eccentricity of any vertex), and *transitivity* (an elementary measure of local segregation which measures the density of connections between a node's neighbors without bias due to disproportionate influence by low-degree nodes).

### Lesion simulation

To determine whether a particular lesioned network differs significantly from other lesioned networks of the same type, null hypotheses regarding the effect of GM and WM loss effects upon network topology were formulated and tested using a framework inspired by the approach of Alstott et al. (2009), who simulated the effects of lesions located in various regions of the cerebral cortex. Localized area removal was performed by deleting the node and its connections which were associated with a single contiguous anatomic parcel, as defined using methods previously described (Destrieux et al., 2010). Distinct lesions were thus generated by removing a single cortical parcel and the associated node from the brain network, and by assuming that the spatial extent of each lesion was that of the corresponding parcel. This process was repeated 148 times (the number of cortical regions in the parcellation scheme) to create 148 lesioned networks in which exactly one cortical parcel was lesioned. The procedure was repeated for all 110 subjects included in the study. Because lesion configurations were defined using the cortical atlas of Fischl et al. (1999a), and the corresponding location of each lesion was identified in every subject by mapping the lesion configuration from the atlas to each subject's cortical surface using existing FS methodology (Fischl et al., 1999a,b, 2004), each lesion had approximately the same position in every subject. For each lesioned network, the node and connections associated with the lesion were deleted from that subject's cortical network, and the network measures previously described were computed. Network metrics were then averaged over all subjects to obtain the average (i.e., most statistically probable) value of each metric for every simulated lesioned network.

### Statistical significance of node removals

Because the absolute values of network metrics increase monotonically with the number of nodes and edges in a network (Zalesky et al., 2010), it is essential to test for the significance of metric values in the context of an appropriately formulated null hypothesis. The use of statistical significance testing is necessary in this case because network measures can often vary unpredictably as a function of the size and density of individual graphs, such that absolute values of network measures are not always meaningful. For example, to determine whether the average path length in a network is significantly different from that in a population of random networks, the most commonly used model for the null hypothesis is that of a randomly constructed network where the number of edges, nodes in- and out-degrees is equal to that of the network being tested (Sporns, 2011). In the present case, however, because the hypotheses being investigated relate to the differential effects of lesioning upon distinct areas of the brain, the null model was formulated as follows. Let *m* represent a network metric (either segregation or integration, as appropriate). For each *m*, the null hypothesis was formulated as the statement that the value of *m* associated with a lesioned network was drawn from the same distribution as that of the values of *m* associated with the healthy network. First, the values of *m* for each of the 110 healthy (*H*) networks were computed, and their mean μ_*H*_(*m*) and standard deviation σ_*H*_(*m*) were also found. Subsequently, for each parcellated region *k* (*k* = 1, …, *G*, where *G* is the total number of parcels), the node associated with *k* was removed from each subject's healthy network, while leaving the rest of the network intact (i.e., only node *k* was removed). In the following step, the value of *m* for each of the 110 networks which were thus lesioned (*L*) was computed, and the mean μ_*L*_(*m*; *k*) and standard deviation σ_*L*_(*m*; *k*) of *m* over subjects was calculated. To test the null hypothesis for *m*, the difference of means μ_*H*_(*m*) − μ_*L*_(*m*; *k*) was tested for significance via a paired *t* test and the corresponding *p*-value was computed. Multiple comparisons were accounted for via the Bonferroni correction at α = 0.05. By means of the procedure described above, the distribution of metric values over subjects for a certain lesioned network (i.e., for a given value of *k*) was compared against the distribution of metric values over the same subjects for the healthy network.

### Principal component analysis of node removals

To identify such groups of nodes whose removal results in lesions with similar effects upon network metrics, a principal component analysis (PCA) was performed. PCA is a mathematical procedure which uses an orthogonal transformation to convert a set of observations of possibly correlated variables into a set of values of uncorrelated variables called principal components (PCs). Rencher (2002) is an excellent reference where a detailed description of this standard technique for multivariate statistical analysis can be found. Because, in our case, PCA can reveal the internal structure in the set of effects pertaining to node removals, it is an excellent tool for quantifying mutually orthogonal patterns of such effects. The number of PCs is less than or equal to the number of original variables. This transformation can be organized in such a way that the first PC has as high a variance as possible (that is, accounts for as much of the variability in the data as possible), and each succeeding PC in turn has the highest variance possible under the constraint that it be orthogonal to (uncorrelated with) the preceding PCs. The results of a PCA can be analyzed in terms of PC scores (the transformed variable values corresponding to a particular data point) and PC loadings (the weight by which each standardized original variable should be multiplied to get the PC score). Here, each subject represents an observation while each ROI represents a variable; consequently, a key strength of PCA which is very useful in this study is that plotting PC factor loadings allows one to map across the cortex groups of parcellated regions which are similar to each other from the standpoint of integration or segregation measures. PCA was chosen instead of other possibly useful methods such as independent component analysis (ICA) primarily because the former method is more parsimonious from a methodological standpoint. Additionally, ICA may be disadvantageous when used or interpreted inappropriately, particularly when its constraint of statistical independence between latent variables is problematic to define in a manner which can be justified rigorously from an empirical standpoint (Delac et al., 2006).

For clarity, it should be explained that the dimensionality of the matrix of network metrics is *G* × *G* × *N* (number of nodes by number of nodes by number of subjects), where each entry in this matrix records the effect of removing region *k* (*k* = 1, …, *G*) upon each other region *k*′ (*k*′ = 1, …, *G*, where *k* ≠ *k*′) for each subject from 1 to *N*. To perform a PCA which captures lesion similarities across subjects, the third dimension of this matrix needs to be collapsed across; one manner in which this can be done is by means of computing the Pearson product-moment correlation coefficient *r* for each pair of regions *k* and *k*′ across subjects. The correlation coefficient is useful here due to its ability to convey the similarity between the effect of removing one node and the effect of removing some other node. Calculation of the correlation coefficient over subjects using the three-dimensional matrix of network metrics generates a two-dimensional matrix of size *G* × *G*, which contains *r* (*k*, *k*′) in each entry, and to which PCA can be applied.

For all possible combinations of cortical region pairs, the Pearson product-moment correlation coefficient *r* between the values of network metric *m* associated with each pair of cortical regions was computed across subjects. By means of this, a correlation matrix **R** of dimensions *G* × *G* was obtained. Within this correlation matrix, rows and columns were arranged according to each region's location, proceeding in antero-posterior order. Right-hemisphere entries were listed first, followed by left-hemisphere entries. Thus, for example, the first region listed was the transverse frontopolar gyrus and the last one was the occipital pole. This arrangement was implemented to acquire the ability of inspecting the correlation matrix for putative anatomical patterns. Next, to identify portions of the cortex which consist of ROIs whose removal leads to similar effects upon integration and segregation properties, a PCA was performed.

### Comparison to unlesioned network metrics

To investigate the extent to which our analysis approach is novel compared to existing canonical methodologies, we sought to determine whether the results of our systematic lesioning approach can be obtained more parsimoniously simply by computing various other network metrics based on the intact (healthy) network of each patient. To do this, the betweenness centrality, clustering coefficient and eccentricity of every node were computed in each subject's intact network using existing methods (Rubinov and Sporns, 2010; Sporns, 2011). Next, a PCA was performed for each metric to determine the PCs of the healthy networks over the cortex in a manner analogous to that applied in the previous subsection. Finally, the correlation coefficients over subjects between the PCA of the lesioned networks and the PCA of the intact networks were computed for each metric. The reasoning here is that, if the results of our lesioning analysis could be reproduced by simply computing various other metrics based on the healthy networks, the correlation coefficients described should be close to unity.

### Statistical significance of edge removals

In addition to investigating the effects of node removals upon network metrics, the impact of edge removals was also studied. To this end, a multivariate feature vector was first designed to include representative measures which could be computed at the level of the entire graph (namely assortativity, mean characteristic path length, density and transitivity). In the following step, the value of the feature vector was computed for the intact network of each subject. Then, for each subject and every possible connection between any pair of brain parcels, the connection in question was removed from the intact network and the value of the feature vector was re-evaluated. This amounted to the removal of exactly one of *G*(*G* − 1)/2 possible connections (*G* = 165 here because *G* is the total number of parcels) from the intact network of each subject, followed by the evaluation of the feature vector after the removal of that connection. Subsequently, we sought to determine whether the removal of each individual edge from the intact network had a statistically significant effect upon the feature vector. The null hypothesis was formulated as the statement that the feature vectors associated with the lesioned networks of the *N* = 110 subjects were drawn from the same distribution as the feature vectors associated with the intact networks. To test this hypothesis, an analysis of variance (ANOVA) for paired samples (i.e., for two measurements on the same network, before and after lesioning) was performed. In this context, the test statistic is Hotelling's *T*^2^ (see chapter 5 in Rencher, 2002), which can be readily converted to an *F* statistic via *F* = (*N* − *p*)*T*^2^/[*p*(*N* − 1)], where *p* is the number of dependent variables in the feature vector (*p* = 4 here). The *F* statistic is distributed as *F*_*p*, *N*−*p*_, and its cumulative distribution function (cdf) can be denoted by cdf (*F*_*p*, *N*−*p*_). The null hypothesis was rejected whenever the inequality 1 - cdf (*F*_*p*, *N*−*p*_) < α/*m* was satisfied, where *m* is the number of comparisons. Thusly, the Bonferroni correction for multiple comparisons was performed with *m* = *G*(*G* − 1)/2, and a value of α equal to 0.05.

### Multilinear principal component analysis of edge removals

In analogy with the PCA to identify groups of *nodes* whose removal results in lesions with similar effects upon network metrics, we also sought to identify groups of *connections* whose removal leads to similar network changes. In this second case, we have a fourth-order tensor *T* of network metrics which is of size *G* × *G* × *G* × *N*. Specifically, for each connection between region *k* (*k* = 1, …, *G*, first dimension) and some other node *k*′ (*k*′ = 1, …, *G*, second dimension) where *k* ≠ *k*′, each entry in *T* specifies the effect upon node *k*″ (*k*″ = 1, …, *G*, third dimension) of removing the connection between nodes *k* and *k*′. The index into the fourth dimension of *T* represents the subject for whom network metric information is available. As in the case of our node removal analysis, we collapse across the fourth dimension of this tensor by computing the Pearson product-moment correlation coefficient *r* for each pair of regions *k* and *k*′ across subjects. This yields a third-order tensor of size *G* × *G* × *G*, which contains a correlation coefficient in each entry. One difficulty which arises here compared to the case of node removals is that PCA must be applied to a tensor of rank 3 as opposed to a tensor of rank 2 (i.e., matrix). This can be accomplished however using multilinear PCA (MPCA), which is a framework for dimensionality reduction of tensor objects; MPCA performs feature extraction by determining a multilinear projection which captures most of the original tensorial input variation (Lu et al., 2008). Similarly to PCA, MPCA allows one to convert the tensorial set of observations into a set of uncorrelated variables called multilinear PCs (MPCs). The first MPC has as high a variance as possible, and the results of an MPCA can be analyzed in terms of MPC loadings, as in the case of PCA. Plotting the factor loadings of an MPC allows one to isolate the group of brain regions sharing the property that removing connections linking one region in the MPC to the rest of the brain affects the network similarly to removing connections between another region in the MPC and the rest of the brain.

## Results

### Systematic network lesioning

In the context of this study, the connectivity core of the human brain is understood to be the set of WM connections which are globally critical to network structure and properties, and whose removal or injury leads to statistically significant changes in these characteristics. To identify those connections whose removal most influences overall network fidelity, a multivariate statistical analysis was performed as previously described, and its results are shown in Figure 1 using a standard 3D graph representation as well as a connectogram. The removal of the WM connections shown in the figure leads to significant network changes, and the transparency of connectogram links associated with each depicted connection is proportional to the statistical significance of the change prompted by removal of the corresponding connection. Link transparency varies in the connectogram such that more transparent links are associated with smaller *F*-values, and more opaque links are associated with larger *F*-values. Because the multivariate analysis leading to the results in Figure 1 was performed based on feature vectors containing a variety of network topology metrics (see section Network measures), it is important to emphasize that these results implicitly relay information on the relationship between the connectivity scaffold and the topological properties of the nodes which they interconnect.

The results in Figure 1 indicate that connections whose removal results in significant network theoretic changes involve structures in the insula (especially the inferior circular sulcus of the insula), the temporal lobe (polar plane and transverse temporal sulcus, bilaterally) and the parietal lobe (e.g., the connection between the angular gyrus and the posterior occipital sulcus). Similarly important connections between the frontal lobe and other lobes (especially the occipital lobe) are also present in the scaffold, in addition to connections between the cerebellum and the occipital lobe (lingual gyrus), and between non-cortical structures. In this last respect, connections between (A) the hippocampus and the amygdala, (B) the thalamus and the hippocampus, (C) the thalamus and the brain stem, and between (D) the putamen and the pallidum are notable. Whereas the most important connections as quantified via network metrics involve ipsilateral structures, connections between left and right subcallosal gyri and limbic structures are notable exceptions. The FA of scaffold connections is also found to vary across brain connections. Specifically, FA is often found to be (A) high (in red) for connections involving structures located in both hemispheres, non-cerebral structures as well as midline cortex, (B) average (in green) for connections relating occipital regions to the rest of the brain, and (C) low (in blue) for numerous long-rage (i.e., fronto-occipital and fronto-parietal) connections. In the context of this analysis, the connectivity core of the human brain is understood to be the set of WM connections which are globally critical to network structure and properties, and whose removal or injury leads to statistically significant changes in these characteristics.

**Figure 2:**
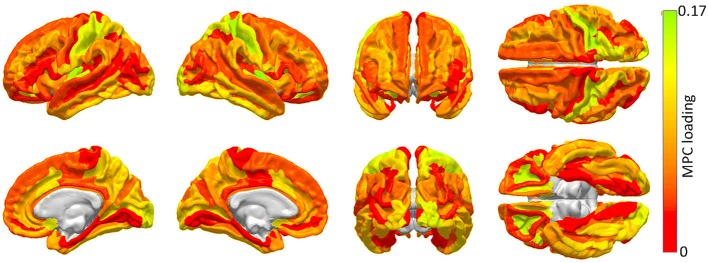
**Cortical areas to which an appreciable proportion of core connectivity scaffold connections are connected**. These regions are identified through a network theoretic application of MPCA, and the factor loadings of the first MPC are mapped over the cortex. Color varies from the minimum to the maximum MPC loading value.

The results of the MPCA to identify cortical regions which are linked by an appreciable proportion of scaffold connections are shown in Figure 2. It should be noted that a detailed discussion of MPCA, accompanied by visual illustrations of multilinear projection, is available in the original publication of Lu et al. (2008), who pioneered the method. However, because understanding these illustrations as well as the technique itself requires delving into considerable mathematical background, we opt to convey instead a simple interpretation of MPCA which conveys the insight which it offers in the context of our study. Firstly, though MPC loadings are analogous to PC loadings, visualization of MPCA results can be more challenging because MPCA involves tensors, which can feature one or more dimensions than PCA. For this reason, we have chosen to display MPC loadings in Figure 2, because these loadings can be straightforwardly mapped onto the cortex in the same manner in which PC loadings are mapped in Figure 4. Additionally, as in the case of PCA, MPCA results can be analyzed in terms of MPC loadings. In the context of our study, suppose that a particular region is assigned a large MPC loading in Figure 2. What this implies is that, when certain connections linking that region to the rest of the brain are removed, the global effect of this removal is similar to that obtained when another region with a large MPC loading is subjected to the same process. Thus, brain networks respond similarly when certain connections which link regions with large MPC loadings to the rest of the brain are removed. In Figure 2, MPCA indicates that the removal of connections between any regions belonging to the first MPC and the rest of the brain has network effects which are similar across MPC member regions. In other words, removing connections linking any MPC member region to the rest of the brain has network effects similar to those of removing connections linking other MPC regions.

The first MPC was found to explain ~10% of the variance in network metrics, which corresponds, in fact, to an appreciable proportion of variance in the context of dimensionality reduction of tensor objects via MPCA (Lu et al., 2008). Subsequent MPCs were found to explain less than 1% of the variance, possibly due to the large variability of network effect patterns which is encountered when individual connections are removed. Because of the low variance explained by these MPCs, their loadings are not shown. Figure 2 highlights brain regions to which a substantial proportion of scaffold connections are connected, specifically primary and secondary visual cortex (calcarine sulcus, cuneus), primary somatosensory cortex (post-central gyrus), anterior cingulate and middle/inferior temporal cortex.

### GM lesion effects upon network properties

**Figure 3:**
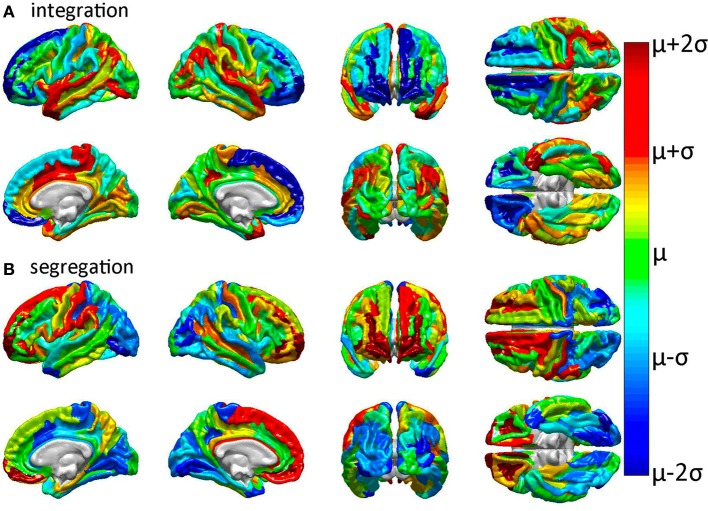
**Relative effects of focal cortical lesioning upon nodal network properties**. Results are displayed for **(A)** integration, as quantified using the characteristic path length, and for **(B)** segregation, as quantified using the average local efficiency. Cortical regions colored in dark blue are regions whose removal is associated with a network property decrease which is lower than the average decrease due to the removal of a randomly selected region. The converse applies to parcels colored in dark red, i.e., these are regions with a higher than expected decrease in the network property due to that structure's removal. The values plotted are the statistical *t* scores for each parcel's integration and segregation measures with respect to the intact (healthy) network. The color displayed for each parcel represents the average *t* score over subjects. Colors range from dark blue (lowest average *t* score over subjects) through green to dark red (highest average *t* score). Parcels drawn in cold colors (blue to green) have mean *t* scores which are lower than the average *t* score over all parcels. Parcels drawn in warm colors (green to red) have *t* scores which are higher than the average *t* score over all parcels.

Previous analyses of GM injury to network performance have shown that GM lesion location and extent affect network properties differentially (Alstott et al., 2009). Here, GM lesion effects were computed and analyzed so as to contrast and compare the WM connectivity scaffold of network edges to the set of prominent GM network nodes (Rubinov and Sporns, 2010; Sporns, 2011). Simulated injuries involving the removal of cortical structures (gyri or sulci) from brain networks were observed to have variable effects upon network properties. Figure 3 displays the effect of each cortical structure's removal upon two of these properties (integration and segregation) compared to their corresponding values in the intact network. As previously noted, integration is quantified here using the characteristic path length, whereas segregation is quantified using average local efficiency.

Inspection of Figure 3 reveals that tight integration of a region within the global network can translate into loose segregation from it, and vice versa. This may reflects what Olaf Sporns (2011) aptly calls “tension between local and global order” in brain networks whereby “segregation and integration place opposing demands on the way in which networks are constructed” [*cf*. Sporns, 2011, (pp. 11–14) for an excellent clarification of this complex and prominent relationship in brain networks]. Given the presence of this effect in the brain, one important advantage of the multivariate statistical analysis performed in our study is that each multivariate feature vector included only uncorrelated network metrics in order to avoid the confounding effect of large covariance between feature variables.

In all cases of structural region removal, network integration was found to be significantly lower after lesioning compared to the healthy network. These results indicate that the superior temporal gyri (bilaterally), the middle cingulate gyrus and paracentral lobule (right hemisphere), and the post-central sulcus (right hemisphere) are particularly sensitive to insult from the standpoint of network integration. On the other hand, two regions which are comparatively less sensitive to injury are the frontomarginal and superior frontal gyri, bilaterally. Collectively, these results confirm and complement previous findings regarding the effects of GM lesions upon network properties (Alstott et al., 2009) and highlight differences between brain regions strongly linked to the connectivity scaffold of WM network edges (Figure 2), as opposed to prominent GM network nodes (Figure 3).

### GM lesion effects upon network topology

Exploring the effect of lesions upon inter-regional connectivity allows one to compare the properties of GM network nodes to those of the WM connectivity scaffold. To accomplish this, the correlation matrix **R** was computed, as described in the previous section. Reiterating, each entry *r*_*ij*_ in **R** represents the correlation coefficient across subjects between the following two quantities. The first quantity is a network measure of interest (either segregation or integration, as in Figure 3) computed after the removal of region *i*; the second quantity is that same measure's value after removal of region *j*. Because **R** is computed over lesioned networks where exactly one region has been removed, **R** can be used to outline anatomical patterns of similarity in how network integration or segregation changes after removing exactly one region. To characterize the structure of **R** in a rigorous fashion, a PCA of it was implemented as described previously. By means of this, we sought to elucidate the extent to which the removal of different cortical regions (i.e., network nodes) might affect the network in a similar way, and to quantify the anatomical pattern of that similarity. This exploration is particularly useful in the context of revealing the complementary relationship between lesion effects to the GM rich club, on the one hand, and to the WM connectivity scaffold, on the other hand. Specifically, the removal of some network node *k* might lead to network changes similar to those prompted by the removal of some other node *k*′. Thus, it would be useful to distinguish between network nodes based on this similarity so as to determine GM areas which respond similarly to injury and to distinguish them from WM connections with this property.

**Figure 4:**
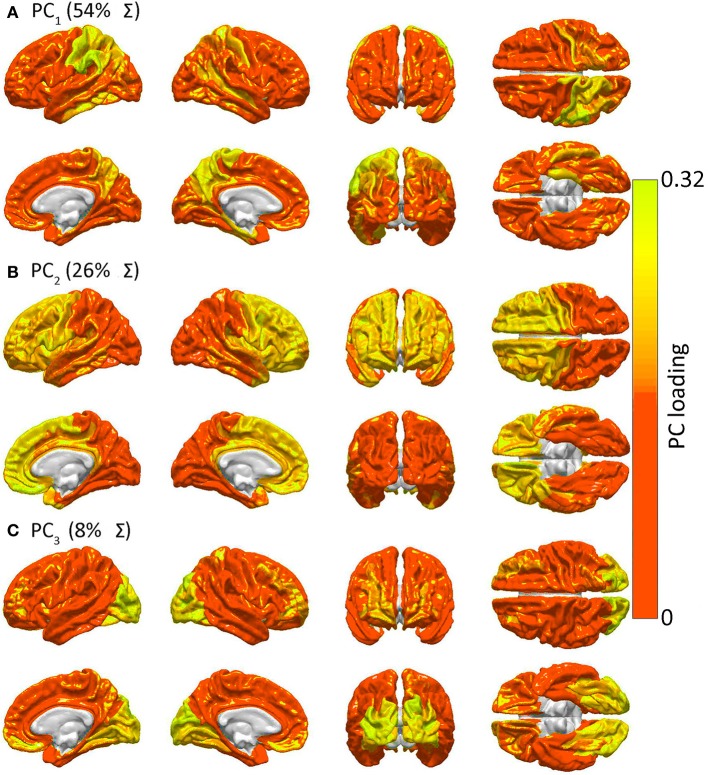
**Similarities in GM lesion effects upon network topology as revealed by PCA**. Shown are lateral, medial, dorsal, ventral, anterior and posterior views of each hemisphere for the first three PCs—i.e. PC_1_ in **(A)**, PC_2_ in **(B)** and PC_3_ in **(C)**—mapped on the cortex, demonstrating the anatomical similarity pattern associated with the extent to which the removal of different regions affects the network in a similar way. For each PC, color varies from the minimum to the maximum value of the PC factor loadings. PC_1_ (54% of the variance Σ in the data) exhibits greater hemispheric asymmetry than the following two PCs and covers the entire left parietal lobe and, to a smaller extent, the left temporal lobe. PC_2_ (26% of Σ) is—by comparison to PC_1_—highly symmetric, and includes the entire frontal lobe of both hemispheres, whereas PC_3_ (8% of Σ) is again symmetric and includes the occipital lobes of both hemispheres.

Figure 4 presents the results of the PCA of **R** to determine spatial patterns of GM lesion effects upon network topology. It should be noted that, while Figure 2 displays regions which are connected to other cortex by *WM connections* whose removal affects global network properties in a similar way, Figure 4 displays groups of *GM regions* whose removal affects global network properties in a similar way. In other words, the two figures both aim to quantify patterns of similarity in how lesioning affects the brain; however, whereas Figure 2 focuses on the effects of *WM* lesions, Figure 4 focuses on the effects of *GM* lesions. The first three PCs are those which explain the largest percentage of the variance Σ (88% in this case) in network integration, and these PCs are mapped over the cortex in Figure 4. For each PC, color varies from the minimum to the maximum value of the PC factor loadings. PCA results indicate the presence of three distinct patterns of network property changes due to network node removals. PC_1_ (0.54 Σ) exhibits greater hemispheric asymmetry than the following two PCs, covering the entire left parietal lobe and, to a smaller extent, the left temporal lobe. PC_2_ (0.26 Σ) is—by comparison to PC_1_—highly symmetric, including the entire frontal lobe bilaterally, whereas PC_3_ (0.08 Σ) is again symmetric and includes the occipital lobes of both hemispheres. Interestingly, although PC_1_ (parietal cortex) covers less area than PC_2_, PC_1_ accounts for the largest proportion of variance. This indicates that lesioning parietal cortex can have the most substantial effect across human subjects from the standpoint of variability in the network measures presented, with individual subjects exhibiting considerably greater changes in network properties than others.

**Figure 5:**
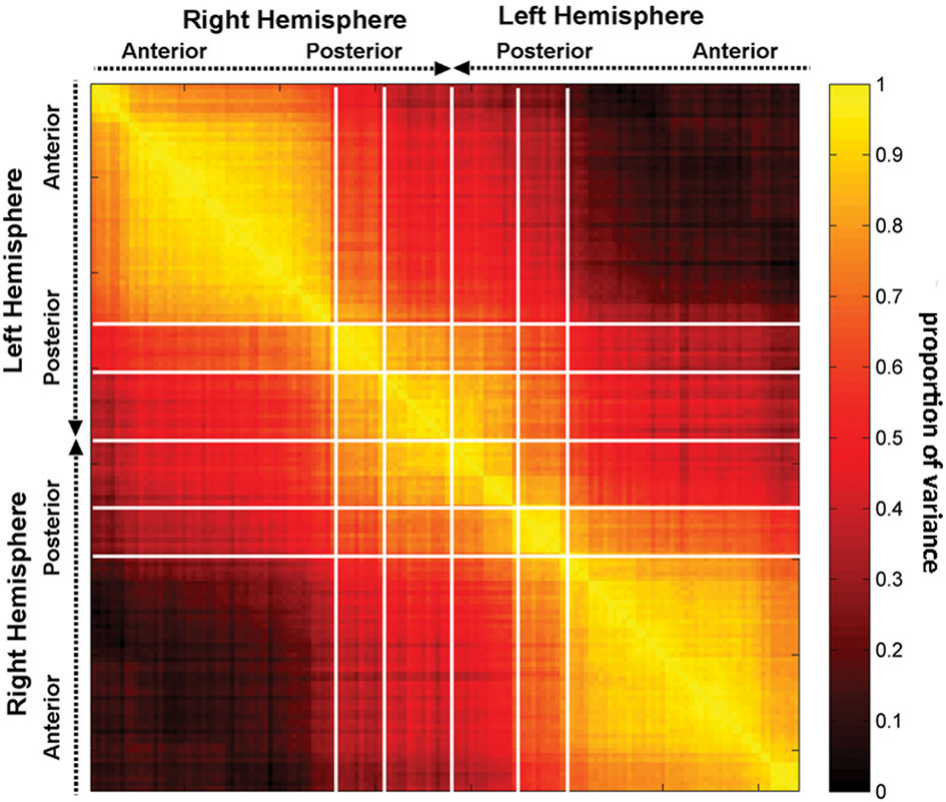
**The correlation matrix R, with each entry *r*_*ij*_ in the correlation matrix representing the correlation coefficient *r* across subjects between two quantities, the first of these being a network measure value (integration in this case) computed after removal of region *i*, and the second one being the value of the same measure after removal of region *j***. Regions are grouped by lobe and displayed within each lobe according to the anatomical location of each region, in antero-posterior order.

Figure 4 indicates that focal injuries can affect brain networks in one of three possible ways, and that each brain region can be classified into one of three groups according to this criterion. Because the cortical patterns depicted in Figures 2, 4 are distinct, these results clearly highlight anatomical differences between the set of prominent GM network *nodes* (Figure 4) and the connectivity scaffold of WM network *edges* (Figure 2). The presence of three blocks within **R** (Figure 5) further confirms the existence of three distinct correlative patterns of network metric changes which occur when a gyrus or sulcus is disconnected from the rest of the brain. The first block along the diagonal of **R** corresponds to PC_2_ and consists of frontal, insular, limbic and temporal areas. The second block corresponds to PC_1_ and contains parietal cortex, and the third block corresponds to PC_3_ and includes gyral and sulcal structures in the occipital lobe. Finally, a correlative comparison between the PCA of the lesioned network and that of the healthy network (see previous subsection) found no correlations greater than *r* = 0.3, which indicates that (A) the results of our analysis cannot be reproduced by simply computing the network metrics of healthy networks, and that (B) the systematic lesioning approach undertaken here cannot be substituted by the calculation of other metrics which were computed based on the healthy networks.

## Discussion

### Significance

In both health and disease, network theory can provide essential insight into the structural properties of brain connectivity (Sporns, 2011), particularly by providing quantitative measures of lesion impact upon neural structure and function, with possible relevance to the prediction of clinical outcome variables and to the task of designing patient-tailored rehabilitation protocols (Irimia et al., 2012a,b). The balance between cortical segregation and integration is of appreciable relevance not only to the study of lesion effects upon the brain, but also to that of brain complexity. Although the brain is functionally segregated at multiple levels of organization, the functions of the former are integrated in both perception and behavior (Tononi et al., 1994). Therefore, it is interesting and illuminating to examine the patterns of network changes in integration and segregation brought about by simulated network lesioning because such alterations affect network structure and implicates functional roles. Although several studies have examined *in silico* the impact of cortical lesions upon network properties as might be measured via functional MRI (Honey and Sporns, 2008; Alstott et al., 2009), our study contributes more substantially to the exploration of lesion impacts upon the core structural network *scaffold*. An important strength of the present study is that it quantifies the connectomic core using a population sample of larger size (*N* = 110) than typically used in connectomic studies (Honey and Sporns, 2008; Alstott et al., 2009).

### Differences between rich club interlinks and core scaffold connections

An important question pertaining to the relationship between rich club and core scaffold connections is whether overlap exists between the sets of edges which define each of these sub-networks of the brain. To clarify this, it is useful to compare our results in our Figure 1 to those in Figure 4 of the article by Van Den Heuvel and Sporns (2011), where rich club connections are mapped. We have opted for a direct comparison between the results of these authors and our own because (i) the results of van den Heuvel and Sporns are based on a finer parcellation scheme than ours, which lends more authority to their findings, and because (ii) the purpose of our present article is to identify the core scaffold rather than to reproduce the previous excellent work of van den Heuvel and Sporns. In their article, these authors identify rich club connections as WM fiber bundles connecting superior frontal and parietal cortices, the precuneus, putamen, hippocampus and thalamus both ipsi- and contra-laterally. In Figure 8 of their paper, the authors depict rich club connections based on a high-resolution cortical parcellation of the cortex, and additionally identify rich club connections between nodes in (what appear to be) the anterior limbic lobe, occipito-temporal junction, and in the lateral and ventral aspects of the inferior temporal lobe. Other rich club connections were also identified, though at brain locations whose precise identity we could not clearly determine due to the connectome visualization method employed by the authors, which does not unambiguously distinguish between all cortical parcels, in contrast to our connectogram approach. Nevertheless, the information provided by van den Heuvel and Sporns in Figures 4 and 8 of their publication remains useful for comparing and contrasting the set of edges which connect rich club nodes, on the one hand, and the core scaffold connections illustrated in Figure 1 of our paper, on the other hand.

Firstly, it is important to note that none of the *inter-hemispheric* connections between the rich club nodes depicted by van den Heuvel and Sporns in Figure 4 of their publication appear in the core scaffold shown in our Figure 1. Furthermore, the only *intra-hemispheric* connection between rich-club nodes which is also present in the core scaffold is a bilaterally occurring connection between the superior frontal gyrus and the precuneus. Secondly, careful comparison of Figure 8D of van den Heuvel and Sporns to our Figure 1 suggests that rich club connections within the limbic lobe occur, with predominance, ipsi-laterally within that lobe and without bilateraly symmetry. By contrast, core scaffold connections involving the limbic lobe are largely bilaterally symmetric, and either (i) inter-hemispheric or (ii) linking the limbic lobe to other lobes ipsi-laterally (see our Figure 1).

With regard to connections between rich club nodes within temporo-occipital areas, we have found it problematic to draw a direct comparison between our results and those of van den Heuvel and Sporns because of the difficulty associated with precisely identifying the locations of rich club nodes in the visual representations of their Figure 8, which makes reliable comparison challenging. It should also be added that, whereas rich club nodes are clearly labeled in their Figure 4, this is understandably not the case in Figure 8 due to the much larger number of cortical regions *G* displayed (82 vs. 1170). A possible remedy to this difficulty which might render the two sets of results more comparable involves refining our own parcellation scheme. Specifically, although the parcellation scheme with *G* = 1170 used by van den Heuvel and Sporns was not available to us for this study, another parcellation scheme with 998 developed by Hagmann et al. is publicly available. Nevertheless, whereas both our parceled regions as well as those in Figure 4 of van den Heuvel and Sporns are based on anatomically-defined structures whose nomenclature is standard in neuroanatomy (Destrieux et al., 2010), more detailed parcellation schemes with large numbers of cortical subdivisions often lack this advantage. In such schemes, only a small subset of parcels are potentially associated with specific labels in standard neuroanatomical nomenclature, which can make specific findings more difficult to situate in the context of mainstream neuroanatomy and consequently more challenging to convey to a broad scientific audience. In addition, as mentioned above, we have found that the presence of a very large number of nodes *G* in the parcellation scheme (998 in the scheme of Hagmann et al. vs. 1170 in that of van den Heuvel and Sporns) makes it difficult to identify the precise locations of rich club nodes relative to anatomical landmarks because other nodes and edges can partially obscure rich club nodes and their connections, even when edge display thresholds are applied (*cf*. Figure 8D in the article of van den Heuvel and Sporns). Incidentally, it is precisely this difficulty which led us to the development of the connectogram visualization, which makes all regions readily visible in two dimensions rather than three (Irimia et al., 2012c). Finally, implementing our study using as many as *G* ≈ 1000 parcels would result in a dramatic increase in the number of statistical comparisons *m* which must be performed because *m* = *G*(*G* − 1)/2. For example, an increase in *G* from 165 to ~1000 would result in an increase in *m* from 13,530 to ~5.0 × 10^5^. This equates to more than a 36-fold increase in the number of statistical comparisons, resulting in a prohibitive increase in the corresponding probability of making type I errors.

For the reasons listed above, we have found that performing our study using 165 anatomically defined regions and relating our results to those in Figure 4 by van den Heuvel and Sporns was more illuminating in terms of comparing our results to theirs. Nevertheless, it is important to acknowledge that parcellation scheme differences between our study, on the one hand, and those of van den Heuvel and Sporns and Hagmann et al., on the other hand, constitute a limitation not only for our study, but also for other studies which aim to compare and interpret network properties in the presence of such differences. Despite such impediments associated with our comparison, the discussion above is sufficient to illustrate the fact that the set of network connections between rich club nodes in the human brain overlap only very moderately—and even then, perhaps accidentally—with the core scaffold described and quantified in the present study. What this suggests is that, although the rich club organization of the human brain remains a useful conceptualization of the human connectome, the core scaffold identified here is a distinct and appreciably valuable set of structural connections whose existence and exploration can have important consequences to future studies of the human connectome, particularly from the standpoint of the connectomic effects of brain trauma.

### Complementarity of rich club and core scaffold

Our results reveal the complementarity between the rich club network of GM *nodes* and the connectivity scaffold of WM *edges* in the human brain. Firstly, our findings on network node properties suggest that brain networks appear to be least affected, from the standpoint of integration, when frontopolar cortex is lesioned, and most affected when the superior temporal lobe, medial limbic, and medial parietal areas are injured. The largest changes in segregation properties are observed when frontopolar and superior frontal cortex are removed, which indicates that these regions contribute appreciably to network segregation in the brain. In frontal lobe injury patients, behavioral and personality changes are common because this lobe has appreciable involvement in cognition, and the extent of damage to frontal cortex is commensurate with dis-inhibition, failure to plan and memory deficiencies (Ietswaart et al., 2008; Zhou et al., 2010). The observation that frontal—particularly frontopolar—lesions cause relatively smaller changes in network integration compared to those due to lesions elsewhere may reflect the fact that an unexpected number of patients whose lesions are exclusively frontal recover reasonably well despite lesion size (Levin et al., 1992), as in the famous cases of Phineas Gage (Van Horn et al., 2012) and E. V. R. (Eslinger and Damasio, 1985). Thus, our finding fits well within the existing medical record, where frontal lobe injuries have been associated with cases of relatively good recovery compared to injuries in other locations. This result is corroborated by those of van den Heuvel and Sporns, who found that frontopolar cortex is topologically distant from primary network hubs and that it is not part of the rich club of vertices in this network (Van Den Heuvel and Sporns, 2011). Incidentally, these same authors found that certain network nodes located in anteromedial frontal and limbic cortex play a prominent role in the rich club network. In our case, we found that (1) anteromedial limbic cortex (particularly in the right hemisphere) has higher-than-average integration, and that (2) frontopolar/superior frontal cortex (particularly in the left hemisphere) has higher-than-average segregation.

The results of this study suggest that (1) the WM connectivity scaffold of network edges has a structural significance which is distinct from, but complimentary to, that of GM rich club nodes, and that (2) the structure of the connectivity scaffold is supported by previous findings on human WM architecture. As pointed out by an anonymous reviewer, comparison of Figures 2, 4A reveals that certain brain regions (e.g., post-central gyrus, cuneus, and precuneus) have similarly large MPC and PC_1_ loadings, respectively. This is indicative of the fact that significant changes are effected upon brain networks both when (A) WM connections linking these regions to other cortex are removed, and (B) when these GM regions themselves are removed. The similarity between Figures 2, 4A is not surprising given that occipital and parietal regions tend to show greater connectivity in humans (Hagmann et al., 2008), and that sensorimotor cortex is involved in the rich-club network (Van Den Heuvel and Sporns, 2011). In our study, the angular and superior temporal gyri of both hemispheres were found to produce drastic changes in network integration properties when lesioned. This finding may also be partly explained by the fact that the superior temporal gyrus is the locus of primary sensory functions such as auditory and olfactory processing, which are critical to the survival of the individual. Thus, the findings suggest that the brain is more sensitive to injuries which affect the loci of basic sensory functions, and more resilient to injuries which affect regions involved in higher functions, such as the frontal pole area. This conclusion is confirmed by the observation that the segregation properties of the cortex are least affected by injuries to the occipital lobe, which is the locus of the primary and secondary visual cortex (Figure 3). Additionally, as Figure 2 indicates, the occipital lobe is part of the WM connectomic core of the human brain, as is sensorimotor cortex. Thus, it is possible that the removal of occipital regions alters segregation patterns so considerably not only because of these areas' involvement in the rich-club network (Van Den Heuvel and Sporns, 2011) but also because the WM connectomic core connects these areas to others.

### Brain network vulnerability to injury

The results presented in Figures 4, 5 reveal how various brain areas can be grouped together based on their vulnerability to injury. Both correlation analysis and PCA show that, based on the criterion of either network integration or segregation, the cortex can be divided into three major areas which have distinct integration properties. Interestingly, these regions are found to overlap, to some extent, with the lobar structures of the brain, namely temporo-parietal cortex (PC_1_), fronto-temporal cortex (PC_2_), and occipital cortex (PC_3_). Because the functions of these cortical areas are distinct (non-visual sensory processing, executive function, and visual processing, respectively), our results lead to the hypotheses that (1) a close relationship may exist between the network properties of each portion of the brain listed above and its functional role, and (2) the response of various brain regions to injury as quantified from the standpoint of network integration/segregation may be predictable based on their function. Arguments in favor of these hypotheses may be found in our previous study (Irimia et al., 2012a), where the specific structural deficits of affected patients were used to relate neuroimaging findings to the existing body of research on the functional roles of the cortical structures affected. Notably, however, the cortical maps and results of Figures 4, 5 are clearly distinct from those of Figures 1, 2, which underlines the distinctiveness between the rich club of network nodes and the connectivity scaffold of network edges.

### Evolutionary considerations

In an evolutionary sense, the accelerated volumetric expansion of the frontal lobe compared to other parts of the neocortex is relatively recent (Semendeferi et al., 2002). In this respect, one of our findings is that lesioning the frontal pole area leads to changes in network integration which are significantly smaller than those caused by other lesions. This allows us to position our study in relation to a previous hypothesis (Kaiser and Varier, 2011) according to which a relationship may exist between the evolutionary history of this structure and the network effects of lesioning it. Such a possibility can, perhaps, be further appreciated in light of the fact that the frontal lobe is heavily involved in high-level cognitive and social functions (Alvarez and Emory, 2006; Amodio and Frith, 2006); this is particularly the case in higher primates including humans, where such functions are essential for social interaction (Adolphs, 2001) in contrast to maintenance of physiological homeostatis, etc. Furthermore, it has been observed (Kaiser and Varier, 2011) that, in phylogenetically newer species, brain regions which are evolutionarily older tend to contain more highly connected nodes, and intersubject variability in connectivity has been significantly correlated with the degree of evolutionary cortical expansion (Mueller et al., 2013). Thus, whereas there may not be a straightforward relationship between network integration changes due to injury and these structures' evolutionary history, it remains conceivable that evolutionary factors may play a role in determining regional vulnerability to injury.

### Limitations and caveats

The results determined here are based on a computational analysis of adult brains, and our findings may not be easily extensible to the brains of children and adolescents. It has long been appreciated (Vargha-Khadem et al., 1994) that focal lesion effects in children are often less pronounced than in adults, possibly due to the relative plasticity of intact regions of the developing brain, which are better able to compensate for damaged regions. In addition, WM connectivity in children is known to differ from that in adults in important ways (Paus et al., 2001), and it can be expected that the results of this study would be different if the methodology were to be applied to a cohort of (pre-) adolescent subjects. Another limitation is that cortical networks were constructed by equating each parcel to a node, and each WM connection to an edge, resulting in connectivity matrices with 165 nodes. It is conceivable that our results could differ to some extent if more granular structural networks were used. Nevertheless, the spatial resolution in our study is comparable to that of other computational neuroimaging studies (Hagmann et al., 2010; Rubinov and Sporns, 2010; Sporns, 2012) and further research is thus necessary to determine the extent to which increasing spatial resolution of models such as ours can affect the results of simulated lesion studies, either qualitatively or quantitatively. Additionally, we acknowledge that the presence of post-lesional experimental data would be immensely beneficial for verification of our simulations and for validation of the translational impact of our study. The intrinsic limitations of deterministic tractography as an evolving methodology for the analysis of DTI volumes should also be acknowledged, given that this technique is not fully reliable for the purpose of accurately capturing structural connectivity, particularly between different areas of the cortex.

## Conclusion

It is important to emphasize that the role played by the rich club of network nodes within the connectome is distinct from, but complementary to, that of the connectivity scaffold. To the best of our knowledge, this is the first study to conceptualize the connectivity scaffold of brain networks as being complementary to other underlying patterns, including the set of rich club nodes. Because the network effects of removing individual WM connections are investigated here systematically, this study reveals the structure of the brain connectivity scaffold at a previously unavailable level of detail. This singular result is of appreciable relevance to ongoing efforts to understand the structure of the human connectome because it sheds light upon the important role played by brain WM network edges, which complement the rich club of cortical network nodes. One potential translational application of this study is that it furthers the goal of developing methods based on connectomics and network theory for the personalized investigation of lesion profiles in individual patients. Such applications may be very useful to the task of determining how specific connectivity scaffold changes due either to gross pathology or to longitudinal WM atrophy can accumulate and ultimately produce appreciable neurological and cognitive deficits in TBI patients. Our study also sheds light upon the extent to which TBI, MS, stroke or cortical resection can differentially affect the network properties of the brain from the standpoints of integration and segregation. Thus, because this study quantifies the WM scaffold of the brain in addition to the differential vulnerability of brain areas to injury, this work can be of substantial relevance to clinicians and basic neuroscientists alike.

## Author contributions

Both authors designed and implemented the study, performed analyses, interpreted the results and wrote the paper.

### Conflict of interest statement

The authors declare that the research was conducted in the absence of any commercial or financial relationships that could be construed as a potential conflict of interest.
